# Data Sharing and Reanalyses Among Randomized Clinical Trials Published in Surgical Journals Before and After Adoption of a Data Availability and Reproducibility Policy

**DOI:** 10.1001/jamanetworkopen.2022.15209

**Published:** 2022-06-02

**Authors:** Damien Bergeat, Nicolas Lombard, Anis Gasmi, Bastien Le Floch, Florian Naudet

**Affiliations:** 1 Department Digestive Surgery, CHU Rennes, Pontchaillou Hospital, Rennes, France; 2 Rennes 1 University, Rennes, France; 3 Institut NuMeCan (Nutrition Metabolism and Cancer), INSERM 1241, Rennes, France; 4 Université de Rennes, CHU Rennes, INSERM, CIC 1414 (Centre d’Investigation Clinique de Rennes), Rennes, France; 5 Université de Rennes, CHU Rennes, INSERM, IRSET (Institut de recherche en santé, environnement et travail)–UMR_S 1085, Rennes, France

## Abstract

**Importance:**

Clinical trial data sharing holds promise for maximizing the value of clinical research. The International Committee of Medical Journal Editors (ICMJE) adopted a policy promoting data sharing in July 2018.

**Objective:**

To evaluate the association of the ICMJE data sharing policy with data availability and reproducibility of main conclusions among leading surgical journals.

**Design, Setting, and Participants:**

This cross-sectional study, conducted in October 2021, examined randomized clinical trials (RCTs) in 10 leading surgical journals before and after the implementation of the ICMJE data sharing policy in July 2018.

**Exposure:**

Implementation of the ICMJE data sharing policy.

**Main Outcomes and Measures:**

To demonstrate a pre-post increase in data availability from 5% to 25% (α = .05; β = 0.1), 65 RCTs published before and 65 RCTs published after the policy was issued were included, and their data were requested. The primary outcome was data availability (ie, the receipt of sufficient data to enable reanalysis of the primary outcome). When data sharing was available, the primary outcomes reported in the journal articles were reanalyzed to explore reproducibility. The reproducibility features of these studies were detailed.

**Results:**

Data were available for 2 of 65 RCTs (3.1%) published before the ICMJE policy and for 2 of 65 RCTs (3.1%) published after the policy was issued (odds ratio, 1.00; 95% CI, 0.07-14.19; *P* > .99). A data sharing statement was observed in 11 of 65 RCTs (16.9%) published after the policy vs none before the policy (risk ratio, 2.20; 95% CI, 1.81-2.68; *P* = .001). Data obtained for reanalysis (n = 4) were not from RCTs published with a data sharing statement. Of the 4 RCTs with available data, all of them had primary outcomes that were fully reproduced. However, discrepancies or inaccuracies that were not associated with study conclusions were identified in 3 RCTs. These concerned the number of patients included in 1 RCT, the management of missing values in another RCT, and discrepant timing for the principal outcome declared in the study registration and reported in the third RCT.

**Conclusions and Relevance:**

This cross-sectional study suggests that data sharing practices are rare in surgical journals despite the ICMJE policy and that most RCTs published in these journals lack transparency. The results of these studies may not be reproducible by external researchers.

## Key Points

**Question:**

What is the association of the implementaton of the International Committee of Medical Journal Editors (ICMJE) data sharing policy with data sharing practices and data availability in the 10 leading surgical journals publishing randomized clinical trials?

**Findings:**

This cross-sectional study of 65 RCTs published before and 65 RCTs published after the ICMJE data sharing policy found no association between the policy and data sharing in the journals studied.

**Meaning:**

This study suggests that most randomized clinical trials published in the 10 leading surgical journals lack transparency and that their results may not be reproducible by external researchers.

## Teaser

This cross-sectional study evaluates the association of the International Committee of Medical Journal Editors data sharing policy with data availability and reproducibility of main conclusions among leading surgical journals.

## Introduction

Randomized clinical trials (RCTs) are the building blocks of an evidence-based approach to medicine and surgery. These trials are used as trusted sources of evidence when designing clinical practice guidelines. It is therefore expected that their results are trustworthy and that their conclusions can be verified. Randomized clinical trial data sharing (ie, sharing statistical codes, study protocols, and individual participant data) holds promise for enhancing transparency and maximizing the value of research.^1^ Data sharing is supported by patients who also want to share their data in a responsible and secure manner.^2^ In 2018, the International Committee of Medical Journal Editors (ICMJE) required that manuscripts submitted to ICMJE journals as of July 1, 2018, must contain a data sharing statement if they are reporting the results of an RCT. If a trial began enrolling participants on or after January 1, 2019, the article must also include a data sharing plan in the trial’s registration.^3^ The ICMJE considers that “it is an ethical obligation to responsibly share data generated by interventional clinical trials because participants have put themselves at risk.”^4^

Although several studies have investigated the use of data sharing policies in general medical journals (eg, *PLOS Medicine*,^5^ *The BMJ*,^5^ *Annals of Internal Medicine*^6^), data sharing in the surgical community is rare. Although transparency has improved over the past 10 years in surgical journals, data sharing statements are an exception, reaching approximately 3% of published RCTs, mostly in 2018 just after the ICMJE policy was issued.^7^

We therefore designed this pre-post cross-sectional study to explore the association between the implementation of the ICMJE policy and the data availability and reproducibility of the main conclusions among the 10 leading surgical journals. We also sought to describe data sharing practices in the surgical field, as well as barriers to data sharing and reanalyses.

## Methods

The methods were specified in advance and documented in a protocol registered on April 17, 2019.^8^ Analyses of data extracted from RCTs were performed in October 2021, and reanalyzed separately once data were obtained. According to French regulations regarding human-participants research, no ethics committee approval was needed for such a meta-research project because it did not involve the inclusion of new patients. Reporting of our study followed the Strengthening the Reporting of Observational Studies in Epidemiology (STROBE) reporting guideline adapted for cross-sectional studies.^9^

### Eligibility Criteria

We surveyed consecutive publications of RCTs that had been submitted to and published by the 10 leading surgical journals before (prior to July 2018) and after (submitted after July 2018) the adoption of data sharing policies by these journals. The 10 leading journals were defined as the top 10 journals with a predominant surgical topic according to the 2018 journal impact factor used in the Web of Science Journal Citation Report.^10^ The journals were selected independently by 2 of us (D.B. and N.L.) and included *Annals of Surgery*, *JAMA Surgery*, *British Journal of Surgery*, *Journal of Thoracic and Cardiovascular Surgery*, *Journal of the American College of Surgeons*, *Surgery for Obesity and Related Diseases*, *Obesity Surgery*, *European Journal of Vascular and Endovascular Surgery*, *Annals of Thoracic Surgery*, and *European Journal of Surgical Oncology*. Consecutive RCTs in these journals published before and after the ICMJE policy were eligible for inclusion whether they were superiority or noninferiority trials. Preliminary reports, secondary analyses, cluster trials, and crossover studies were not eligible and were therefore excluded.

### Search Strategy and Study Selection

Eligible RCTs were identified from PubMed using a standardized search string (eAppendix 1 in the Supplement). Study selection was conducted independently in a blinded, standardized manner by 2 of us (D.B. and N.L.). Assessment for the eligibility of studies was performed in reverse chronological order before the policy and in chronological order after the policy. Studies were included if they corresponded to the inclusion criteria as previously described. Disagreements were resolved by consensus or in consultation with a third reviewer (F.N.).

### Outcomes

The primary outcome was data availability (ie, the receipt of sufficient data to enable reanalysis of the primary outcomes). We followed a standardized procedure to retrieve the data. In cases in which there was a data sharing statement in the published article: (1) if it was stated that the data would not be shared, the data were considered as not available; (2) if the statement described how to retrieve the data, we followed all instructions to retrieve the data. In cases in which the instructions involved emailing the study authors or a data custodian, 3 attempts were made. When there was no data sharing statement, we emailed the corresponding author, and 3 attempts were made. If there was no response to these emails, the same procedure was repeated with a second author among the leading authors (first, last, or secondary). In this case, we explained that even if the article was not eligible for the ICMJE policy, we were interested in reanalyzing their data (the standard letter is provided in eAppendix 2 in the Supplement). No specific funding from the reanalysis team was devoted to database collection. If authors asked for funding for this purpose, we asked what the amount of money involved was and recorded the amount, but the procedure was stopped and the data were considered not available.

The secondary outcomes concerned the description of (1) data sharing intention (ie, type of data sharing plan, material shared, and explicit reasons for not sharing); (2) data sharing modalities (ie, time lapse for data availability, reason for nonavailability when authors initially intended to share their data, deidentification issues [concerning name, date of birth, or address], type of data set shared, and availability of statistical codes); and (3) reporting of key features for reproducibility (conformity of eligibility criteria with the first study registration, details of setting and locations where data were collected, detailed description of the intervention to enable replication, conformity of the primary outcome with the first study registration with reasons for nonconformity, and conformity of the secondary outcomes with the first study registration with reasons for nonconformity).

The reproducibility of the results was explored for trials sharing their data. One of us (D.B.) performed the same analyses as described in the published report of the study. If insufficient information was provided in the study report, we contacted trial investigators to obtain the necessary clarifications. The reanalyses concerned only the primary outcome (or outcomes, if there were several) for each trial. The effect sizes and their 95% CIs for each primary outcome reanalyzed were reported, as well as the *P* values. This investigation aimed to assess whether, on the basis of both quantitative (effect sizes and *P* values) and qualitative (clinical judgment) considerations, any discrepant results in the reanalysis led to a different conclusion from the one reported in the original publication. After this assessment procedure, we classified the studies into the following 4 categories: (1) fully reproduced, (2) not fully reproduced but same conclusion, (3) not reproduced and different conclusion, and (4) not reproduced (or partially reproduced) because of missing information. We planned a specific procedure to double-check results that were flagged as not reproduced (eAppendix 3 in the Supplement).

We qualitatively explored some common challenges that were encountered during this research. Among these challenges, we noted whether the sharing of data and/or codes required clarifications for which additional queries had to be made to the authors, to obtain the relevant data and/or codes, to clarify their labels and/or use, and to reproduce the original analysis of the primary outcomes. A classification of these clarifications and of all challenges encountered was created, grouping similar clarifications for descriptive purposes.

### Data Extraction

A standardized data collection sheet was developed to extract RCT characteristics and outcomes. Data collection was conducted independently in a blinded, standardized manner by 2 of us (N.L. and B.L.F.). Disagreements were resolved by consensus or in consultation with a third reviewer (F.N.).

### Sample Size Calculation

Based on early reports of RCT data sharing, we hypothesized that the rate of data availability among RCTs before the policy was implemented would be low. This hypothesis was subsequently confirmed in a scoping review.^11^ Furthermore, we hypothesized that the increase in the rate of data availability would range from less than 5% to more than 25%, a somewhat large but expected increase after the implementation of the policy. With an α risk of .05 and a power of 90%, we estimated that 65 RCTs published before the data sharing policy was implemented and 65 RCTs published after the data sharing policy was implemented (130 in total) were necessary to assess whether data availability increased after the policy was implemented.

### Changes to the Initial Protocol

Modification of the initial protocol was necessary to ensure that the RCT selection strategy was based on submission dates when available (in addition to publication dates) to identify RCTs submitted and published after the ICMJE policy as accurately as possible. This modification of the submission date was made to avoid as much as possible the inclusion of studies published after July 2018 but submitted well before this date and therefore not eligible for the new policy. Using logistic regression, we also explored whether the presence of a data sharing statement was associated with the date of publication.

After data collection, we described the journals’ data sharing policies in their instructions to authors (ie, no policy, less stringent policy than the ICMJE policy, or policy compliant with the ICMJE policy) (1) before data collection, 6 months after the policy in December 2018, and (2) after data collection, in September 2021 at the time of reporting our study.

### Statistical Analysis

Analyses were performed using R statistical software, version 3.6.3 (R Group for Statistical Computing). Outcomes were compared before and after the policy was implemented. Continuous data were presented using median values and IQRs and compared with the Mann-Whitney test. Categorical data were presented as percentages and compared with the χ^2^ test or a 2-sided Fisher exact test when χ^2^ test application conditions were not met. Odds ratios are presented when necessary. Two-sided statistical significance was set at *P* < .05.

Concerning reanalyses, we used the statistical methods implemented in the original articles to compute the *P* values of our reanalysis. Effect sizes were expressed using Cohen *d* (with 95% CIs) or incidence rate ratios for count outcomes. The meta package^12^ and the metacont function were used. In some cases, when results were presented as median (IQR) values in the original publication, we assumed a normal distribution (mean = median and SD = IQR/1.35). We adopted the same approach and conversion for our reanalysis (as well as the same rounding of values). Because this is a strong assumption, our analysis of reproducibility also involved a comparison of median values and IQRs.

## Results

### Characteristics of RCTs Included

**Figure 1.**
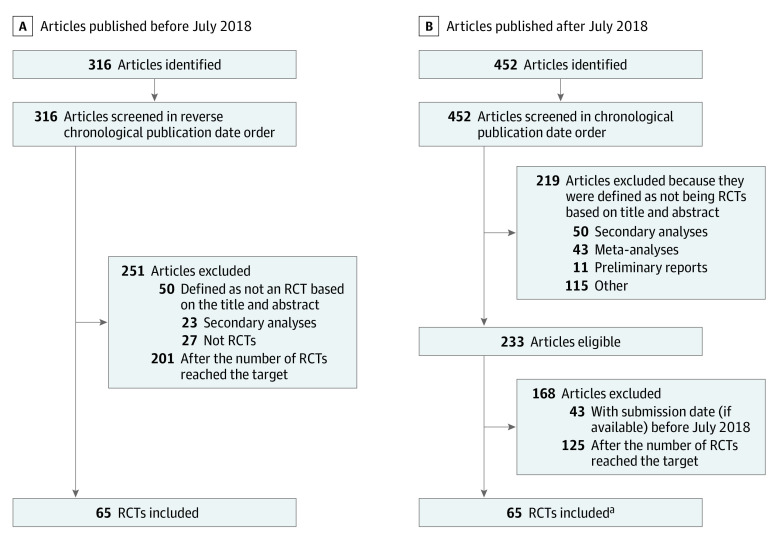
Selection of the Randomized Clinical Trials (RCTs) Included ^a^Two RCTs were identified a posteriori as having been submitted before July 1, 2018.

A total of 130 RCTs (65 published before the policy was implemented and 65 after the policy was implemented) were included (Figure 1). Their main characteristics are detailed in Table 1. These studies were mostly about digestive surgery (n = 83 [63.8%]). A total of 111 studies (85.4%) were prospectively registered, and 117 (90.0%) reported a conflict of interest disclosure. Statistically significant differences were evidenced before and after the policy was implemented in the numbers of RCTs published in each journal, the types of design, and the comparators. For studies published after the ICMJE requirement, the submission date was available for only 24 RCTs. We found a posteriori that 2 of the studies included were submitted before the ICMJE requirement (the first on January 25, 2018,^13^ and the second on June 28, 2018^14^). These studies were retained in the analysis because they were published after the ICMJE policy was implemented. The presence of a data sharing statement was not associated with the publication date for studies published after the policy was implemented (eFigure in the Supplement).

**Table 1.** Characteristics of Randomized Clinical Trials Included

Characteristic	ICMJE data sharing policy, No. (%)		*P* value
	Before (n = 65)	After (n = 65)	
Journal			
* Annals of Surgery*	17 (26.2)	20 (30.8)	.04
* Annal of Thoracic Surgery*	9 (13.9)	1 (1.5)	
* Bristish Journal of Surgery*	14 (21.5)	8 (12.3)	
* European Journal of Surgical Oncology*	1 (1.5)	1 (1.5)	
* European Journal of Vascular and Endosvascular Surgery*	4 (6.2)	1 (1.5)	
* JACS*	5 (7.7)	6 (9.2)	
* JAMA Surgery*	3 (4.6)	8 (12.3)	
* Obesity Surgery*	10 (15.4)	13 (20.0)	
* Surgery for Obesity and Related Diseases*	2 (3.1)	4 (6.2)	
* Journal of Thoracic and Cardiovascular Surgery*	0	3 (4.6)	
Surgical topic			
Digestive	40 (61.5)	43 (66.2)	.36
Urology	1 (1.5)	0	
Vascular	4 (6.2)	5 (7.7)	
Thoracic	5 (7.7)	6 (9.2)	
Anesthesia	9 (13.9)	6 (9.2)	
Gynecology	0	3 (4.6)	
Others	6 (9.2)	2 (3.1)	
Continent of the corresponding author			
Asia	15 (23.1)	9 (13.9)	.40
Europe	32 (49.2)	33 (50.8)	
North America	14 (21.5)	13 (20.0)	
Oceania (Australia and New Zealand)	1 (1.5)	2 (3.1)	
Middle East	1 (1.5)	2 (3.1)	
Africa	2 (3.1)	2 (3.1)	
South America	0	4 (6.1)	
Comparators			
Drug	9 (13.9)	16 (24.6)	.03
Device	15 (23.1)	12 (18.5)	
Surgical technique	14 (21.5)	24 (36.9)	
Path of care	26 (40.0)	12 (18.5)	
Surgical formation	1 (1.5)	1 (1.5)	
Blinding			
Open label	33 (50.8)	30 (46.2)	.60
Single blinded	18 (27.7)	16 (24.6)	
Double blinded	14 (21.5)	19 (29.2)	
Type of blinding not detailed	2 (3.1)	0	.50
Study design			
Superiority	50 (76.9)	61 (93.9)	.01
Noninferiority	15 (23.1)	4 (6.2)	
Design not described	21 (32.3)	9 (13.9)	.02
Sample size, median (IQR), No. of participants	104.0 (67.0-245.0)	145.0 (100.0-215.0)	.12
Primary outcome clearly defined			
Yes	55 (84.6)	56 (86.2)	>.99
No	10 (15.4)	9 (13.9)	
Positive study on the primary outcome			
Yes	33 (50.8)	28 (43.1)	.47
No	29 (44.6)	33 (50.8)	
Type of sponsor			
Public or academic	21 (32.3)	14 (21.5)	.57
Private, laboratory, or industry	11 (16.9)	8 (12.3)	
Nonprofit sector or charity	8 (12.3)	11 (16.9)	
Mixed	4 (6.2)	7 (10.8)	
No information	16 (24.6)	21 (32.3)	
No funding	5 (7.7)	4 (6.2)	
Private sponsorship			
No	37 (56.9)	32 (49.2)	.83
Provided device	3 (4.6)	1 (1.5)	
Provided intervention	1 (1.5)	1 (1.5)	
Provided drug	1 (1.5)	4 (6.2)	
Provided partial financial support	2 (3.1)	2 (3.1)	
Provided total financial support	4 (6.2)	3 (4.6)	
Not detailed	1 (1.5)	1 (1.5)	
Clinical trial registration			
Yes	55 (84.6)	56 (86.2)	>.99
No	10 (15.4)	9 (13.9)	
COI disclosure			
Yes	56 (86.2)	61 (93.9)	.24
No	9 (13.9)	4 (6.2)	

Abbreviations: COI, conflict of interest; ICMJE, International Committee of Medical Journal Editors; *JACS*, *Journal of the American College of Surgeons*.

### Data Sharing Intentions and Modalities

**Figure 2.**
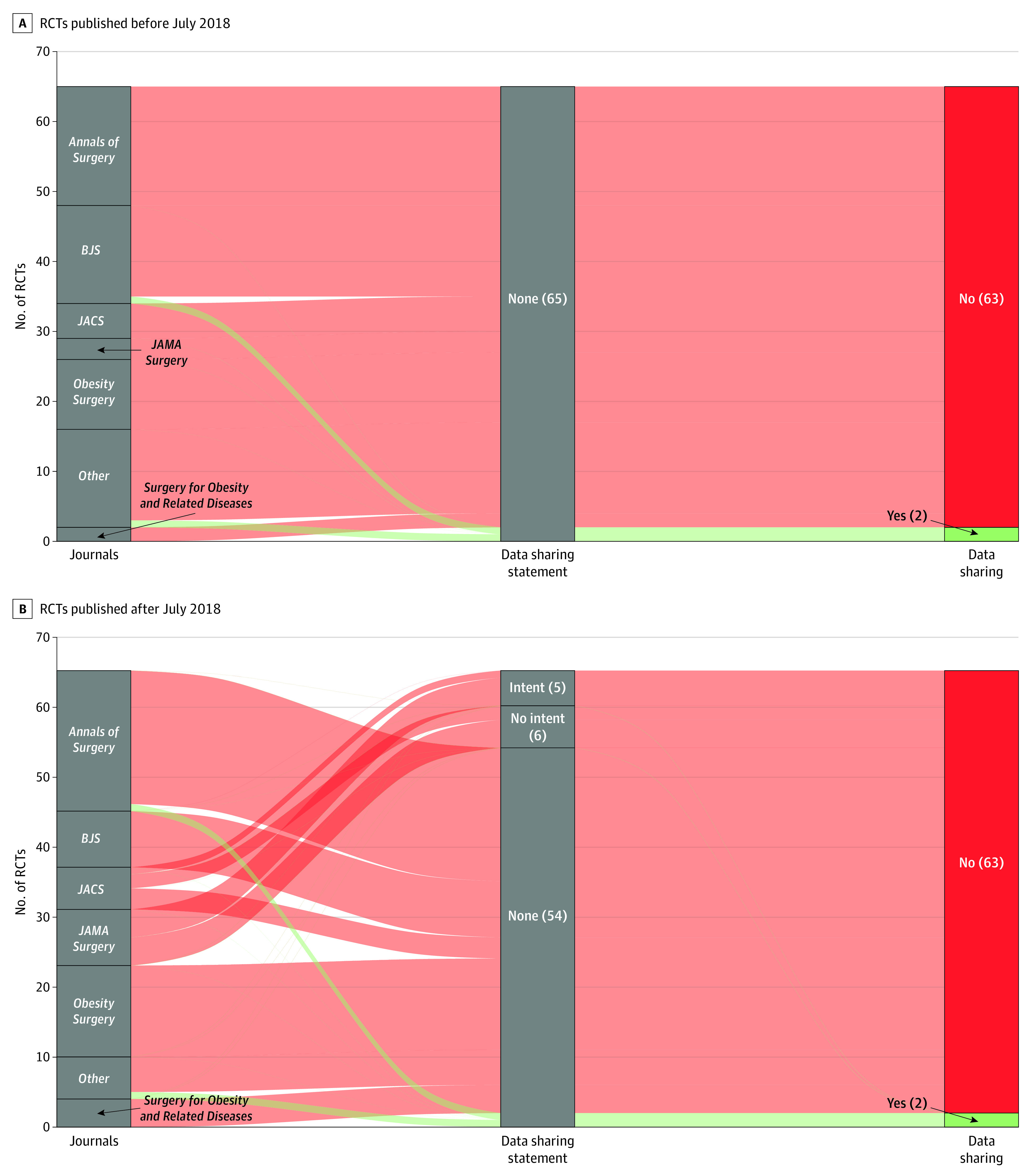
Existence of Data Sharing Statements and Data Sharing Before and After the Implementation of the International Committee of Medical Journal Editors (ICMJE) Policy A, Randomized clinical trials (RCTs) published before the ICMJE data sharing policy. B, RCTs published after the ICMJE data sharing policy. The green alluvial pattern of lines indicates data finally shared. In the lower part of each panel, a comparison of the data sharing statement rate and the actual data sharing rate between the 2 periods is detailed. *BJS* indicates *British Journal of Surgery*; and *JACS*, *Journal of the American College of Surgeons*.

The existence of data sharing statements and data availability according to journal are illustrated in Figure 2. After the ICMJE policy, a data sharing statement was detailed for 11 of 65 RCTs (16.9%) vs none before the policy (risk ratio, 2.20; 95% CI, 1.81-2.68; *P* = .001). When a data sharing statement was detailed, 5 of 11 RCTs (45.5%) expressed the intent to share data, while 6 of 11 (54.5%) stated that data were not available. For all 5 studies with an intent to share data, the sharing modality was “upon request” by emailing an RCT team member. Among these studies, no correspondence could be established despite a strict application of the procedure detailed in the data sharing statement. Among the remaining 119 RCTs that did not include a data sharing statement (65 before and 54 after the policy), we obtained at least 1 response for 19 RCTs (16.0%). Among those 19 studies, 11 declined to share the data (5 for lack of ethics approval, 3 because of a lack of understanding of our project, 1 because of lack of time, and 2 because they were already sharing their data for an individual participant data meta-analysis with another team), and 4 initially agreed to share the data but never actually shared it. Data availability, our primary outcome, was met for 4 of 130 studies (3.1%),^15,16,17,18^ with 2 of 65 studies (3.1%) published before the ICMJE policy and 2 of 65 studies (3.1%) published after the ICMJE policy (odds ratio, 1.00; 95% CI, 0.07-14.19; *P* > .99). The time lapse for obtaining RCT data was 3.5 weeks and 8.5 weeks, respectively, for the 2 studies published before the policy and 4 weeks for the 2 studies published after the policy.

### Reproducibility and Key Features for Reproducibility

**Figure 3.**
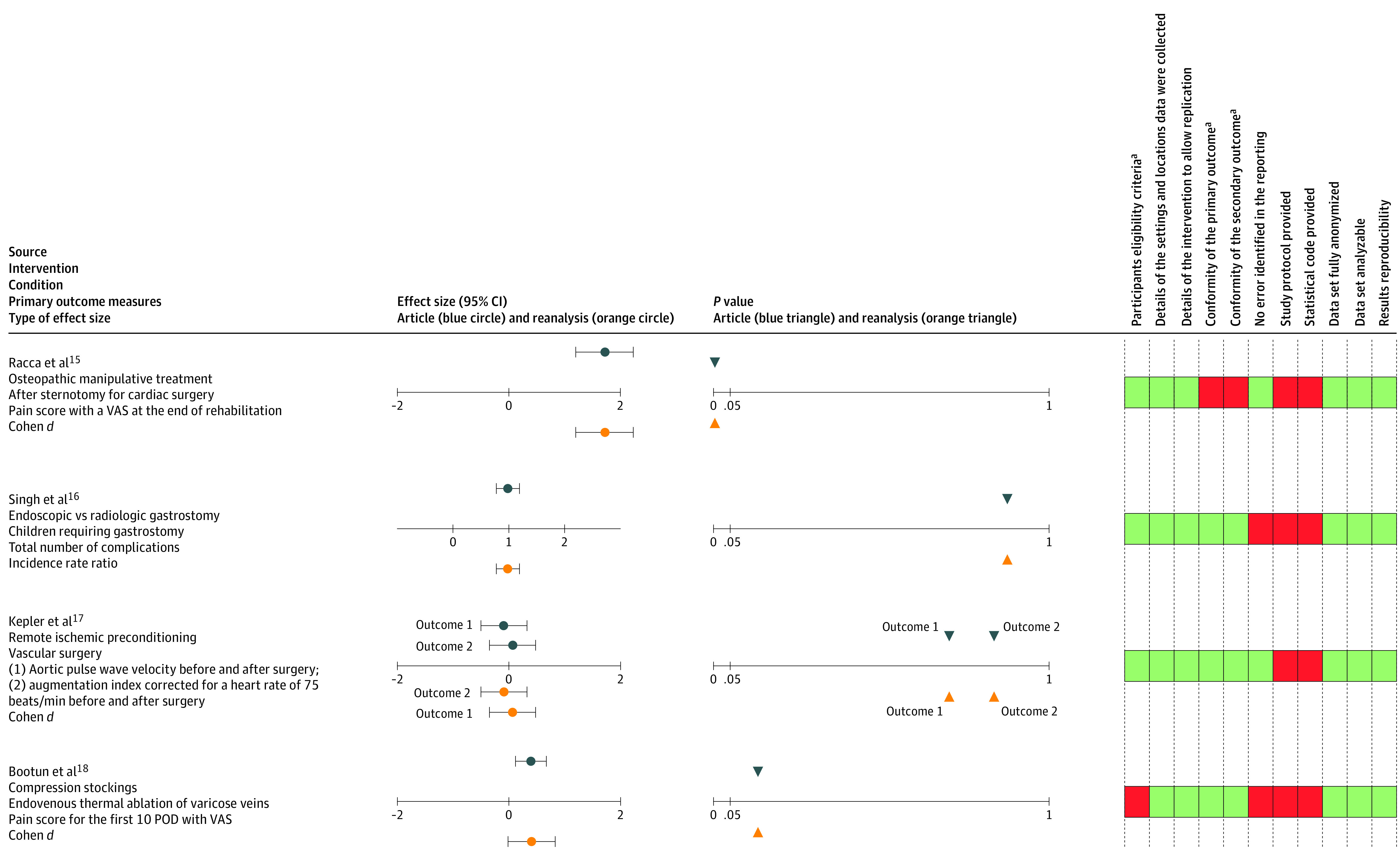
Results of Reanalyses and Key Features for Reproducibility POD indicates postoperative day; VAS, visual analog scale. ^a^Compared with study registration.

Results of the reanalyses and information about key features for reproducibility are detailed in Figure 3.^15,16,17,18^ The data sets obtained (n = 4) were analyzable in all cases and were abstracted (ie, restricted to primary outcome and group allocation only) in 1 case. In no case did the author provide us with the study protocol or the statistical analysis code. All shared data sets were deidentified for patients’ names, birth dates, and addresses.

For 1 study,^18^ we had to contact authors for further details concerning the method used in their analyses (ie, whether the authors used a specific method to deal with missing data, which concerned 115 of 203 patients). The authors clarified our query and answered that only the 88 patients with available data were analyzed. For the second study,^16^ we wanted to discuss an error identified in the reporting because there were 192 patients with data to allow the primary outcome analysis vs 193 patients in the published article. No answer was provided to our request for clarification.

Despite minor numerical inconsistencies (Figure 3; eTable in the Supplement), the results were considered fully reproduced for all 4 studies in terms of effect sizes and *P* values. Concerning the reproducibile features, discrepancies between the clinical registration and the final report were identified in 2 of 4 studies.^15,18^ The first concerned participant exclusion criteria (“presence of C6 disease” was not mentioned in the registration on ClinicalTrials.gov), and the second concerned the timing of outcome measures, planned at 21 days in the clinical registration and measured at the time of rehabilitation in the final report.

### Common Challenges

The most common challenge that we faced was the communication with study authors (when requesting data and performing reanalyses). Data sharing statements relying on a single corresponding author were a problem because sometimes the email addresses were simply invalid. In addition, the lack of metadata and other study documents made it harder to reanalyze the studies. The lack of anticipation of data sharing in the conduct of certain studies resulted in consent and ethical issues compromising the scope of the data sharing.

### Evolution in Data Sharing Policies During the Study Period

In December 2018 (before data collection), 6 journals had a data sharing policy, but this policy was less stringent than the official ICMJE requirement for 5 of 6 journals, and only 1 journal’s policy was ICMJE compliant. In September 2021 (after data collection), 7 journals had a data sharing policy (6 less stringent than the official ICMJE requirement and 1 that was ICMJE compliant). The results are summarized according to journal in Table 2.

**Table 2.** Evolution of Journal Policies Before and After Data Collection According to Their Official Instructions for Authors

Journal	ICMJE affiliation^a^	Before data colletion (December 2018)		After data collection (September 2021)	
		Data sharing policy	Type of policy compared with ICMJE requirements	Data sharing policy	Type of policy compared with ICMJE requirements
*Annals of Surgery*	Yes	No	Not available	No	Not available
*Annals of Thoracic Surgery*	Yes	No	Not available	No	Not available
*Bristish Journal of Surgery*	Yes	Yes	Less demanding	Yes	Less demanding
*European Journal of Surgical Oncology*	Yes	Yes	Less demanding	Yes	Less demanding
*European Journal of Vascular and Endosvascular Surgery*	Allusion	Yes	Less demanding	Yes	Less demanding
*JACS*	Yes	No	Not available	No	Not available
*JAMA Surgery*	Yes	Yes	Compliant	Yes	Compliant
*Obesity Surgery*	Yes	Yes	Less demanding	Yes	Less demanding
*Surgery for Obesity and Related Diseases*	Allusion	Yes	Less demanding	Yes	Less demanding
*Journal of Thoracic and Cardiovascular Surgery*	Yes	No	Not available	Yes	Less demanding

Abbreviations: ICMJE, International Committee of Medical Journal Editors; *JACS*, *Journal of the American College of Surgeons*.

^a^ Information extracted from instruction for authors on journals’ website; “Allusion” indicates that the journal is not listed on the ICMJE website as an affiliated journal but that references to ICMJE guidelines are made in the instruction for authors.

## Discussion

### Statement of Principal Findings

Among the 10 leading surgical journals, this pre-post cross-sectional study found no association between the implementation of the ICMJE policy and data sharing. Data sharing statements appeared after the policy in these journals, but their implementation in published RCTs was insufficient and highly variable from one journal to another. Only *JAMA Surgery* had a policy in line with ICMJE requirements, although the policy was announced in 2017^19^ and came into effect on July 1, 2018. Other surgical journals had less stringent policies. As a consequence, few studies had data sharing statements, while almost half expressed an intention to share. However, no data set was retrieved for any of the studies expressing an intention to share data. The few available data sets were retrieved from RCTs published without data sharing statements. In our study, when data were available, reanalyses based on the approach of the original authors made it possible to reproduce results on the primary outcomes, with minor discrepancies in all cases. Nevertheless, in 2 cases, our reanalyses required clarifications from the study authors (and in 1 case we received no response).

Evidence is accumulating to support the fact that statements on data availability are poorly implemented^20^ and not effective. For instance, for more than 10 years (2007-2017) of a policy implementing data sharing statements, no association was identified between data sharing intent and data reuse in RCTs published in the *Annals of Internal Medicine*.^6^ Mandatory policies could be more efficient. Still, in 2 journals that adopted mandatory policies (*The BMJ* and *PLOS Medicine*), data were available for reanalyses of only half the publications.^5^ Of course, data sharing requests for the purpose of a reanalysis are less likely to be honored than those related to any other secondary use, such as individual participant data meta-analyses.^21^

Our study illustrates the common practical challenges faced by data sharing initiatives^22^; authors are difficult to contact, the process of data sharing itself is time-consuming and resource-consuming, it requires an adequate technical infrastructure, and it requires legal and ethical support. All these barriers could be addressed preemptively, at an early phase in the study process; when designing the study, it is indeed possible (1) to request appropriate funding, (2) to obtain appropriate ethics approval, (3) to inform study participants correctly, and (4) to provide an appropriate environment supporting data sharing. Instituting this process implies major changes in our research culture.

Beyond the data sharing challenge, we identified some recurrent transparency issues regarding the RCTs analyzed. Primary outcomes were not clearly defined in 15% of the studies, and 28% of the studies had no information about funding sources. The role of journals in the promotion of transparency by way of strong policies should not be underestimated in improving value and reducing waste in research.^7,23^ New indicators that valorize research transparency are being developed to incentivize journals to develop and adequately implement these optimal policies. For instance, the “TOP factor”^24^ is being developed by the Center of Open Science to assess journals for transparency and openness.^25^

### Perspectives

Our results are in line with a growing literature^20,22,26^ demonstrating that the ICMJE data sharing policy has not yet succeeded in making data available from RCTs. Even data on transparency and on data sharing practices or the promotion of data sharing in the surgical community are scarce.^7,27,28^

Obviously, journals are not the only, nor the best, leverage to ensure data sharing. The impetus could be provided on several levels, such as via patients,^2^ researchers, funders, and universities. If most biomedical science faculties currently use traditional metrics, such as productivity metrics, for hiring, promotion, and tenure,^29^ there is a strong incentive to shift from productivity to reproducible research practices, such as data sharing.^30,31^ However, such new policies must have an evaluation component, and meta-research efforts are needed to provide evidence on these policies in terms of increased transparency and reproducibility.

### Limitations

This study has some limitations. It does not reflect what is practiced in all surgical journals because we limited the study to a small sample of journals selected on the basis of their journal impact factor. However, important surgical RCTs are published in these journals, and, in the context in which journals are evaluated according to their impact factors, these journals are often considered as forerunners for their editorial practices and are liable to instigate changes in the norms. In addition, our study does not reflect all RCTs performed in the surgical field because some of them are published in general medicine journals, such as the *New England Journal of Medicine*, *The Lancet*, or *JAMA*. In these journals, data sharing statements are appropriately implemented, but data availability is still problematic.^20,26^

This pre-post cross-sectional study may have been performed too early after the implementation of the ICMJE policy. The time to implementation of other research transparency criteria, such as compliance with the Consolidated Standards of Reporting Trials (CONSORT) 2010 statement for publishing RCTs, was quite long.^32^ Perhaps the change resulting from the ICMJE data sharing statement will require time before becoming a success. As journal policies are liable to evolve in the future, there is a need to continuously monitor and update our findings. Adding a plan to our protocol to contact the institution in the case of no response from a corresponding author could have increased our data sharing rate for reanalyses.

We aimed to include RCTs published after the ICMJE policy, and we also tried to exclude, when possible, studies submitted before the policy was implemented. Unfortunately, details of the submission step dates were rarely available in the articles or on PubMed. Some of the articles included may have been submitted just before the ICMJE policy was implemented, and it is possible that editors applied the policy only for articles submitted after the policy was implemented. We explored the possibility of a bias of this type but found no association between implementation of a data sharing statement and the date of the publication after the policy, suggesting that it was a minor issue.

In our assessment of the few trials sharing their data, we found no differences in conclusions pertaining to treatment decisions, but this result should not be overinterpreted. We explored only a small aspect of reproducibility, namely, inferential reproducibility (ie, different scientists analyzing the same data set come to similar conclusions),^33^ and we adopted the approach used by the authors. In 1 specific case,^18^ we had doubts concerning the management of missing values, and the authors clarified that they used no imputation method to perform their analyses. This clarification enabled the reproduction of the negative results observed on the primary outcome. Still, with more than 50% of missing data, this analysis can hardly be considered as an intention-to-treat analysis. Any imputation method could have resulted in different estimates in terms of effect size. In most cases, there were small differences in terms of effect size, suggesting that there is still room for improvement.

## Conclusions

This pre-post cross-sectional study suggests that most RCTs published in surgical journals lack transparency, and, consequently, their results were not reproducible by external researchers. Our results challenge the effectiveness of the ICMJE data sharing policy as implemented by surgical journals, and a call for action toward the adopting stronger policies is required.

## Notes

Supplement. **eAppendix 1.** Standardized Search String **eAppendix 2.** Standard Letter for Data Sharing Solicitation **eAppendix 3.** Specific Procedure to Double-Check Results That Would Have Been Flagged as not Reproduced During the Reanalysis Phase of the Study **eFigure.** Data-Sharing Statement Evolution Since Implementation of the ICMJE Data Sharing Policy **eTable.** Exact Numbers of Effect Size With 95% CI Extracted From Papers and Calculated From Reanalyses
