# Supplementary material for: Data Sharing and Reanalyses Among Randomized Clinical Trials Published in Surgical Journals Before and After Adoption of a Data Availability and Reproducibility Policy
Source: JAMA Netw Open. 2022 Jun 2;5(6):e2215209. doi: 10.1001/jamanetworkopen.2022.15209 (PMC9163999; doi:10.1001/jamanetworkopen.2022.15209)
Supplement: Supplement. — eAppendix 1. Standardized Search String eAppendix 2. Standard Letter for Data Sharing Solicitation eAppendix 3. Specific Procedure to Double-Check Results That Would Have Been Flagged as not Reproduced During the Reanalysis Phase of the Study eFigure. Data-Sharing Statement Evolution Since Implementation of the ICMJE Data Sharing Policy eTable. Exact Numbers of Effect Size With 95% CI Extracted From Papers and Calculated From Reanalyses [file jamanetwopen-e2215209-s001.pdf]

Supplementary Online Content

Bergeat D, Lombard N, Gasmi A, Le Floch B, Naudet F. Data sharing and reanalyses among randomized clinical trials published in surgical journals before and after adoption of a data availability and reproducibility policy. *JAMA Netw Open.* 2022;5(6):e2215209. doi:10.1001/jamanetworkopen.2022.15209

eAppendix 1. Standardized Search String
eAppendix 2. Standard Letter for Data Sharing Solicitation
eAppendix 3. Specific Procedure to Double-Check Results That Would Have Been Flagged as not Reproduced During the Reanalysis Phase of the Study
eFigure. Data-Sharing Statement Evolution Since Implementation of the ICMJE Data Sharing Policy
eTable. Exact Numbers of Effect Size With 95% CI Extracted From Papers and Calculated From Reanalyses

This supplementary material has been provided by the authors to give readers additional information about their work.

© 2022 Bergeat D et al. *JAMA Network Open.*

eAppendix 1. Standardized Search String

Before the intermediate date point

“Annals of surgery”[Journal] OR “JAMA surgery”[Journal]) OR “The British journal of surgery”[Journal]) OR “The Journal of thoracic and cardiovascular surgery”[Journal]) OR “Journal of the American College of Surgeons”[Journal]) OR “Surgery for obesity and related diseases : official journal of the American Society for Bariatric Surgery”[Journal]) OR “Obesity surgery”[Journal]) OR “European journal of vascular and endovascular surgery : the official journal of the European Society for Vascular Surgery”[Journal]) OR “The Annals of thoracic surgery”[Journal]) OR “European journal of surgical oncology : the journal of the European Society of Surgical Oncology and the British Association of Surgical Oncology”[Journal]) AND (“2016/07/01”[PDAT] : “2018/07/01”[PDAT]) AND Randomized Controlled Trial[ptyp]

After the intermediate date point

“Annals of surgery”[Journal] OR “JAMA surgery”[Journal]) OR “The British journal of surgery”[Journal]) OR “The Journal of thoracic and cardiovascular surgery”[Journal]) OR “Journal of the American College of Surgeons”[Journal]) OR “Surgery for obesity and related diseases : official journal of the American Society for Bariatric Surgery”[Journal]) OR “Obesity surgery”[Journal]) OR “European journal of vascular and endovascular surgery : the official journal of the European Society for Vascular Surgery”[Journal]) OR “The Annals of thoracic surgery”[Journal]) OR “European journal of surgical oncology : the journal of the European Society of Surgical Oncology and the British Association of Surgical Oncology”[Journal]) AND (“2018/07/01”[PDAT] : “present”[PDAT]) AND Randomized Controlled Trial[ptyp]

© 2022 Bergeat D et al. *JAMA Network Open.*

eAppendix 2. Standard Letter for Data Sharing Solicitation

Dear Dr XXXX,

I'm Dr Bergeat Damien, working as a surgeon in the University Hospital of Rennes in France. With one of my colleague, Florian Naudet, we are interested in the impact of the ICMJE data sharing policy on data's availability and on the reproducibility of primary findings in the leading surgical journals. Regarding this, we have decided to realize a survey of data sharing before and after the implantation of ICJME data sharing policy on the 1st of July 2018. For more information, our study is registered here XXXX and the protocol details are available following this link: XXXXX)**.**

As your study 'XXXX' recently published in 'XXXX" is exhibiting data from a randomized clinical trial,

We would greatly appreciate if you could share with us the obtained data (with explained labels for each variable) and any analysis code that might be relevant.

Best regards,

Damien Bergeat MD,
University of Rennes 1, Rennes, France
Institut NuMeCan, INRA 1341, INSERM 1241, Université de Rennes 1
Nutrition Gut Brain (NGB)

Phone : +33 6 76 65 35 24

ORCID : orcid.org/0000-0002-7743-1146

Web-sites :
https://www.researchgate.net/profile/Bergeat_Damien

e-mails :
damien.bergeat@gmail.com
damien.bergeat@chu-rennes.fr

Florian Naudet, MD, PhD,
University of Rennes 1, Rennes, France
Clinical Investigation Center (INSERM 1414) and Adult Psychiatry Department, Rennes University Hospital, Rennes, France

Phone : +33 6 80 36 26 79

ORCID : orcid.org/0000-0003-3760-3801

Web-sites :
https://metrics.stanford.edu/about-us/bio/florian-naudet-0
https://www.researchgate.net/profile/Florian_Naudet
https://publons.com/author/922954/florian-naudet#profile

e-mails :
floriannaudet@gmail.com
florian.naudet@chu-rennes.fr

© 2022 Bergeat D et al. *JAMA Network Open.*

eAppendix 3. Specific Procedure to Double-Check Results That Would Have Been Flagged as not Reproduced During the Reanalysis Phase of the Study

Any disagreement or uncertainty over such conclusions were resolved by consulting a third coauthor with expertise in both clinical medicine and statistical methodology (FN). After this assessment process, if it was determined that the results (and eventually conclusions) were not reproduced, the statistician will independently reanalyze the data to confirm such a conclusion. Once the “not reproduced” status of a publication had to be confirmed we planned to contact the authors of the study to discuss the source of the discrepancy.

© 2022 Bergeat D et al. *JAMA Network Open.*

eFigure. Data-Sharing Statement Evolution Since Implementation of the ICMJE Data Sharing Policy

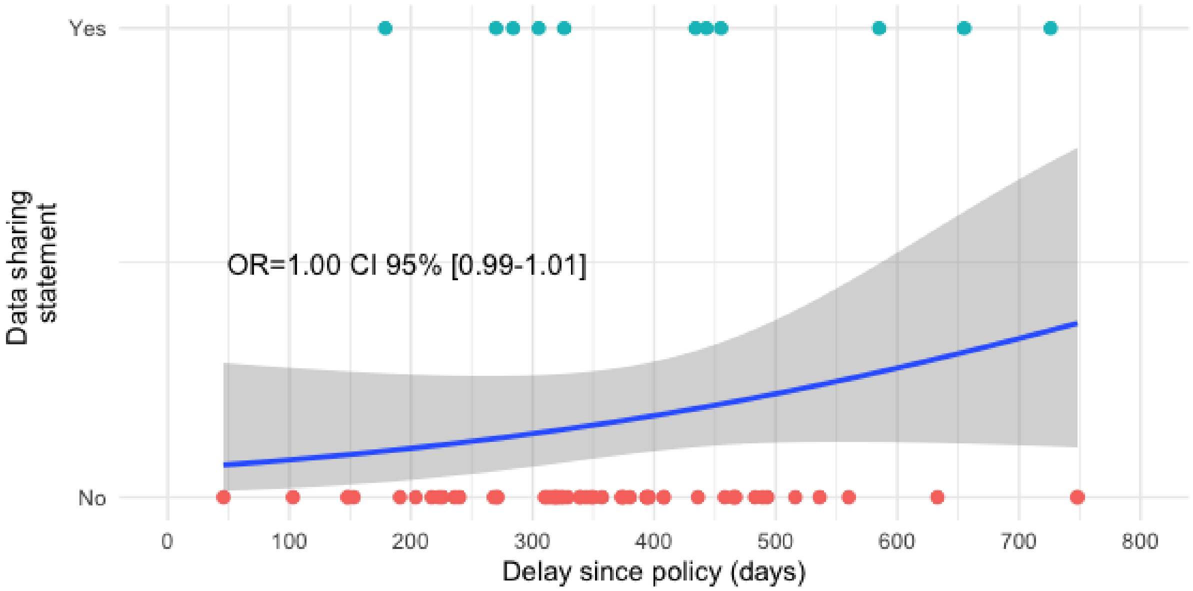

© 2022 Bergeat D et al. *JAMA Network Open.*

eTable. Exact Numbers of Effect Size With 95% CI Extracted From Papers and Calculated From Reanalyses

Supplementary Table 1: Exact numbers of Effect Size with 95% CI extracted from papers and calculated from re-analyses (as a complement to the Figure 3)

Study Ref, Intervention, condition	Primary outcome measures	Type of effect size	Effect size [95%IC] paper	Effect size [95%IC] re analyze
Ref 14, Osteopathic manipulative treatment, Post sternotomy for cardiac surgery	Pain score with a visual analog scale at the end of rehablitation	Cohen d	1.7093 [1.1973; 2.2214]	1.7093 [1.1973; 2.2214]
Ref 15, Percutaneous endoscopic versus radiologically inserted gastostomy, Children requiring gastrostomy	Total number of complication	Incidence rate ratio	0.98 [0.80-1.21]	0.98 [0.80; 1.21]
Ref 16, Remote ischaemic preconditioning, Vascular surgery	Aortic pulse wave velocity before and ater surgery	Cohen d	-0.0913 [-0.5048; 0.3222]	-0.0913 [-0.5048; 0.3222]
	Augmentation index corrected for a heart rate of 75 bpm before and ater surgery	Cohen d	0.0634 [-0.3500; 0.4768]	0.0658 [-0.3476; 0.4792]
Ref 17, Postoperative compression stockings, Endovenous thermal ablation of varicose veins	Pain score for the first 10 postoperative days with a visual analog scale	Cohen d	0.3884 [0.1114; 0.6655]	0.4035 [-0.0195; 0.8266]

© 2022 Bergeat D et al. *JAMA Network Open.*
